# First evidence of hemiclitores in snakes

**DOI:** 10.1098/rspb.2022.1702

**Published:** 2022-12-21

**Authors:** Megan J. Folwell, Kate L. Sanders, Patricia L. R. Brennan, Jenna M. Crowe-Riddell

**Affiliations:** ^1^ School of Biological Sciences, The University of Adelaide, Adelaide, SA 5005, Australia; ^2^ Department of Biological Sciences, Mount Holyoke College, South Hadley, MA 01075, USA; ^3^ School of Agriculture, Biomedicine and Environment, La Trobe University, VIC 3086, Australia; ^4^ Museum of Zoology, University of Michigan, Ann Arbor, MI 48108, USA; ^5^ Ecology and Evolutionary Biology, University of Michigan, Ann Arbor, MI 48109, USA

**Keywords:** clitoris, intersex, hemipenes, DiceCT, histology, squamate

## Abstract

Female genitalia are conspicuously overlooked in comparison to their male counterparts, limiting our understanding of sexual reproduction across vertebrate lineages. This study is the first complete description of the clitoris (hemiclitores) in female snakes. We describe morphological variation in size and shape (*n* = 9 species, 4 families) that is potentially comparable to the male intromittent organs in squamate reptiles (hemipenes). Dissection, diffusible iodine contrast-enhanced micro-CT and histology revealed that, unlike lizard hemiclitores, the snake hemiclitores are non-eversible structures. The two individual hemiclitores are separated medially by connective tissue, forming a triangular structure that extends posteriorly. Histology of the hemiclitores in Australian death adders (*Acanthophis antarcticus*) showed erectile tissue and strands/bundles of nerves, but no spines (as is found in male hemipenes). These histological features suggest the snake hemiclitores have functional significance in mating and definitively show that the hemiclitores are not underdeveloped hemipenes or scent glands, which have been erroneously indicated in other studies. Our discovery supports that hemiclitores have been retained across squamates and provides preliminary evidence of differences in this structure among snake species, which can be used to further understand systematics, reproductive evolution and ecology across squamate reptiles.

## 1. Introduction

Genitalia are some of the fastest evolving characteristics in amniotes with internal fertilization [1]. In these taxa, comparative studies of genitalia provide insights into the role of sexual selection in speciation and the evolution of reproductive traits [2]. Unfortunately, studies of female genitalia have lagged next to an overwhelming focus on male genitalia across amniotes [1,3,4]. This is despite some evidence that female genitalia, and the clitoris in particular, have a key functional role in reproduction [5–8]. For example, variation in clitoris morphology has been linked to different degrees of sexual arousal that could lead to increased reproductive fitness by enticing females to copulate or forming social bonds. Increasing vaginal lubrication, relaxing the vaginal opening and preparing the reproductive tract to receive sperm are among other potential functions of the clitoris [8–11].

Studies on the male hemipenes in lizards and snakes are extensive (e.g. [12]), and have fundamentally shaped ideas on the shared developmental origins of the phallus in amniotes (e.g. [13]), systematic controversies, sexual conflict (e.g. [14]) and diversity of sexual characteristics within the squamate reptiles (e.g. [14,15]). Similar studies of female hemiclitores are rare, and in fact, it is often assumed that the clitoris is vestigial or lost across lineages of squamates [16]. Even when hemiclitores are described in lizards, these have been hypothesized to provide a stimulatory role for the male during intromission [17], rather than to stimulate the female as is the case in other amniotes [8]. Hemiclitores in lizards are eversible and resemble features of the hemipenes such as the sulcus spermaticus and retractor muscles [17–20].

The apparent lack of a hemiclitores in adult snakes is puzzling because this organ is found in most adult female amniotes with the exception of birds [21,22]. During squamate development, the paired genital buds continue growing to create hemipenes or regress in size to form the hemiclitores [23]. Reports of hemiclitores in adult snakes, however, are either, (i) inappropriate citations of literature that discussed lizards rather than snakes, (ii) different sex genitalia in snakes (e.g. intersex or male hemipenes), (iii) vague descriptions without anatomical references or (iv) confused with adjacent anatomy such as the scent glands (e.g. [24]). Many erroneous reports of hemiclitores actually describe hemipenes from intersex individuals, including *Bothrops insularis*, which have a remarkably high prevalence of intersex individuals with functional oviducts [25], *Bothrops jararaca* [26] and *Lycodryas maculatus* [27]. This confusion may stem from imprecise terminology combined with incomplete examinations of gonad anatomy, as some papers define intersex individuals as ‘females with a hemiclitoris', where the hemiclitores were actually intersex hemipenes, and females as ‘females without a hemiclitoris’ [28,29], while other papers describe intersex individuals as ‘females with hemipenes’ [26,27,30–34]. We reviewed these spurious reports and conflicting descriptions of squamate hemiclitores in [27].

Here, we provide the first macro morphological descriptions of hemiclitores using dissection in seven adult female snakes (Elapidae, Viperidae and Pythonidae) and diffusible iodine contrast-enhanced micro-CT (DiceCT) scanning in three adult female snakes (Elapidae and Colubridae). We selected a focus species, the Australian common death adder (*Acanthophis antarcticus*), to conduct in-depth morphological descriptions of hemiclitores using a combination of dissection, DiceCT scanning and histology. Using histology, we compared hemiclitores structure in females of this species with conspecific male hemipenes from an adult and juvenile. Using DiceCT scanning, we demonstrate the difference between the hemiclitores and the adjacent scent glands, which have previously been erroneously reported as hemiclitores [24]. Clarifying the difference between hemipenes and hemiclitores clears the path for a more comprehensive understanding of snake hemiclitores anatomy and potential function, as well as improving our understanding of intersex genitalia in squamates.

## 2. Materials and methods

### (a) Specimens and euthanasia

We examined female genitalia in 10 adult specimens, eight frozen and two fresh-fixed females, across nine species: *Acanthophis antarcticus*, *Agkistrodon bilineatus, Bitis arietans, Helicops polylepis, Lampropeltis abnorma, Morelia spilota, Pseudechis colleti, Pseudechis weigeli* and *Pseudonaja ingrami*. We also examined the micro-anatomy of the male genitalia in an adult and a juvenile specimen (*Acanthophis antarcticus*) (electronic supplementary material, table S1)*.* The adults were wild caught and were sourced from either Venom Supplies Pty. Ltd., private collections, or the University of Michigan Museum of Zoology (UMMZ). The juvenile *A. antarcticus* was born at Venom Supplies.

Once euthanized via injection of pentobarbitone, the specimens were immediately frozen at −20°C. Adult female, male and juvenile male *A. antarcticus* specimens were used for histology, and an adult female was used for DiceCT scanning (electronic supplementary material, table S1). The adult females of *A. bilineatus, B. arietans, M. spilota, P. colleti, P. weigeli* and *P. ingrami* were used for dissection morphology, and *H. polylepis* and *L. abnorma* were used for DiceCT morphology (electronic supplementary material, table S1).

### (b) Histology

For the female *A. antarcticus*, the tail was dissected dorsally to identify the hemiclitoral structure medial to the two scent glands, posterior to the cloaca. The hemiclitores structure and both scent glands were removed from the tail and fixed in 10% buffered formalin. For both males, the inverted hemipenes structures were removed and preserved in 10% buffered formalin.

The excised genitalia from the *A. antarcticus* histology specimens were processed and stained for paraffin histology. Each sample was sliced longitudinally with a microtome 10 times at 5 µm (first nine slides not stained—45 µm), once at 10 µm, then once again at 5 µm. The slides were stained in haematoxylin and eosin (H&E), Bielschowsky silver and Masson's Trichrome, respectively. The slides were scanned using an Axio Scan.Z1 Automated Slide Scanner (Axioscan, Zeiss, Germany) and the ZEN Blue software version 3.4 (Zeiss Zen blue edition, Zeiss, Germany).

### (c) Diffusible iodine contrast-enhanced micro-CT

The tail of the female *A. antarcticus* was removed with a transverse amputation just above the posterior lip of the cloaca. The tail of the death adder and the two colubrid full snake DiceCT specimens were fixed in 10% buffered formalin, rinsed for 24 h and transferred into 70% ethanol for at least two weeks. The tail and whole-bodied specimens were transferred into 50% ethanol for 48 h, then into 25% ethanol for 48 h before submersing in 1–1.25% Lugol's iodine solution (I_2_ + KI + H_2_O) for approximately 14 days, as per the following protocol for DiceCT [35]. Scanning was conducted on the tail prior to and post-staining using a SkyScan-1276 Micro-CT (Zeiss, Germany) at the University of Adelaide (Aluminium 1 mm filter, 10 µm, 90 kV, 200 µA), and on the whole-bodied specimens on a Nikon Metrology XTH 225ST µCT scanner (Xtect, Tring, UK) at the UMMZ. The two-dimensional tomography slices for each scan were reconstructed in Avizo version 9.2 (Thermo Fisher Scientific, USA) or Volume Graphics Studio Max version 3.2 (Volume Graphics, Heidelberg, Germany) and the hemiclitores segmented using a thresholding tool. The contrast between soft tissue in the tail was low but the hemiclitores could clearly be defined by comparing its position with the images of the dissection and histology and by demarcations between the hemiclitoris and the two scent glands.

## 3. Results

### (a) Discovery of hemiclitores in colubrid, viperid, pythonid and elapid snakes

**Figure 1.**
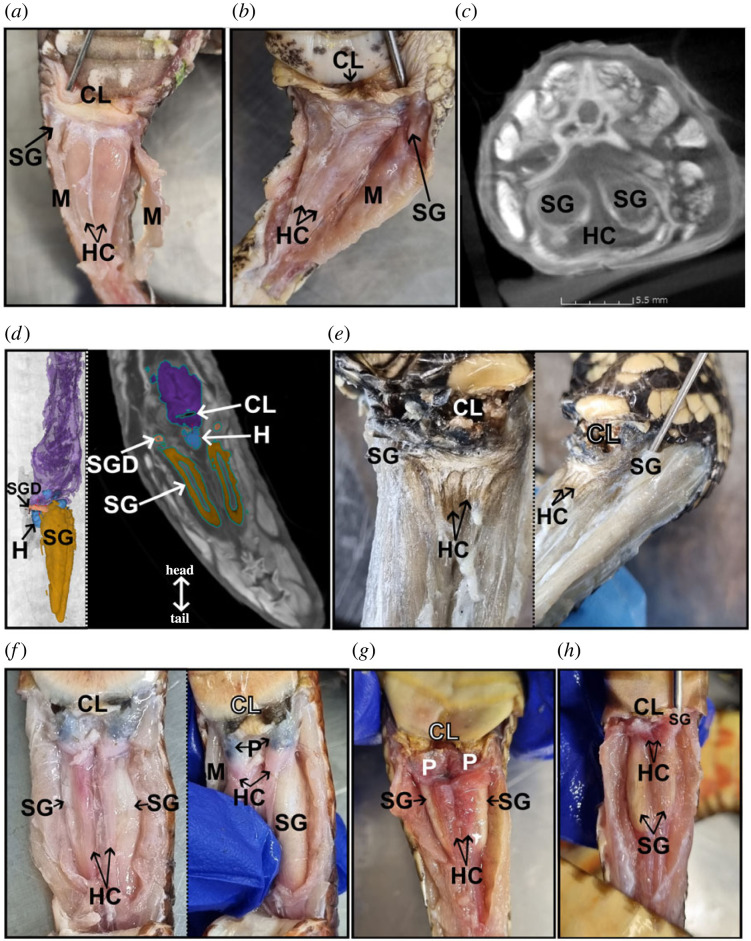
Macroanatomy of the snakes hemiclitores and scent glands in mature female (*a*,*b*) viperid, (*c*,*d*) colubrid, (*e*) pythonid and (*f*–*h*) elapid snakes (specimen IDs and information in the electronic supplementary material, table S1). (*a*) *Agkistrodon bilineatus*. (*b*) *Bitis arietans*. (*c*) Unsegmented DiceCT scan transverse slice of a *Helicops polylepis*. (*d*) DiceCT three-dimensional model (left of dotted line) with ventral view of the two-dimensional segmented CT scan (right of dotted line) of a *Lampropeltis abnorma.* (*e*) Two dissection images of *Morelia spilota* specimen. (*f*) Two dissection photos of *Pseudechis colleti* specimen, undisrupted gross anatomy of the hemiclitores (left of dotted line) and hemiclitores moved to the side to show the scent gland (right of dotted line)*.* (*g*) *Pseudechis weigeli.* (*h*) *Pseudonaja ingrami*. Dotted lines separate two images that are from the same specimen but a different view. **CL**: cloaca; **H** or **HC**: hemiclitores; **M**: muscle; **P:** pockets; **SG**: scent glands; **SGD**: scent gland duct. (Online version in colour.)

**Figure 2.**
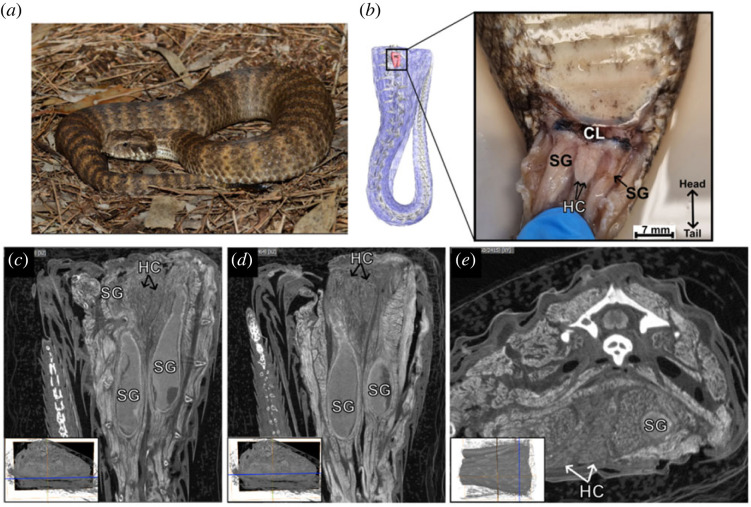
Macroanatomy of two mature female common death adders (*Acanthophis antarcticus*) hemiclitores and scent glands (specimen IDs and information in the electronic supplementary material, table S1). (*a*) Female death adder ‘AA99’ specimen image. (*b*) Ventral view of a DiceCT three-dimensional model of female specimen ‘AA79’ with and dissection of female specimen ‘AA99’. (*c*,*d*) Two ventral view two-dimensional longitudinal slices from a DiceCT scan of a female specimen ‘AA79’ tail (blue line = slice position). (*e*) Transverse two-dimensional DiceCT slice of female specimen ‘AA79’. **CL**: cloaca; **HC**: hemiclitores; **SG**: scent glands. Death adder image credit: Luke Allen.

In all species, the hemiclitores were clearly identified as two separate and non-eversible structures in the tails of females, posterior to the cloaca and medial or medioventral to the two scent glands (figures 1 and 2). DiceCT and dissection revealed the hemiclitores are separated medially by connective tissue that together forms triangular structures, with some shape variation and significant size variation across species (figures 1 and 2). Unlike lizard hemiclitores, all snake hemiclitores examined lacked spines, sulcus spermaticus and retractor muscles, and could not be everted by manual manipulation. Some hemiclitores were large and conspicuous, occupying most of the anterior tail region that extended dorsally towards the spine (*Agkistrodon bilineatus*) (figure 1*a*), whereas others were small and medioventral to the scent gland (*Helicops polylepis*—figure 1*c*; *Pseudonaja ingrami*—figure 1*h*). The elapids and colubrids presented with the smallest hemiclitores, and the viperids had the most prominent ones (figures 1 and 2). Some elapids, *Pseudechis colleti*, *Pseudonaja ingrami* and *Pseudechis weigeli,* presented with hemiclitores that were thin and laid over the top of the scent glands (ventral position) but still in a central position in the tail, thus, medioventral (figure 1*f–h*). However, *Lampropeltis abnorma* (figure 1*d*)*,* like *Acanthophis antarcticus*, presented with small hemiclitores that extended deeper towards the spine than in other elapids. Another cryptic feature found in some species, *Pseudechis colleti* and *Pseudechis weigeli*, was the presence of detached ‘pockets’ anterior to the hemiclitores, posterior to the cloaca and medial to the scent gland openings (figure 1*f,h*). These pockets consisted of two empty soft tissue pouches, separated through the centre, with the opening along the posterior cloaca lip and pouch extending posteriorly towards the hemiclitores. There was no protrusion of pouch/pocket into the hemiclitores, thus the pockets were detached from the hemiclitores.

### (b) Intraspecific comparison of genital micro-anatomy in *Acanthophis antarcticus*

**Figure 3.**
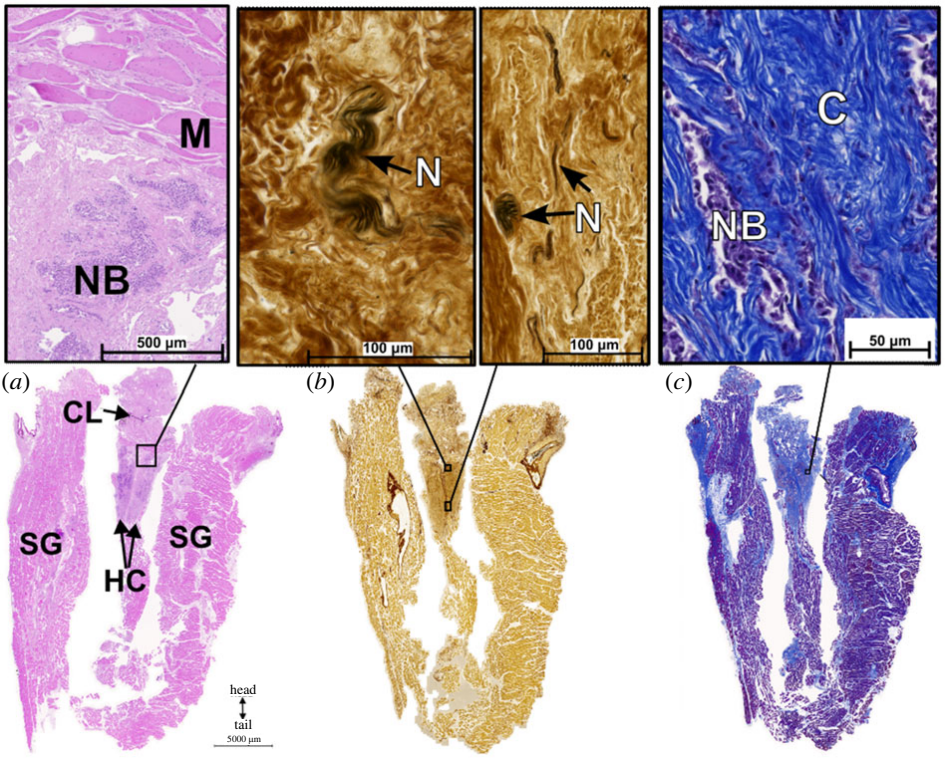
Histology of the hemiclitores and scent glands from mature female death adder specimen ‘AA99’ with (*a*) hematoxylin and eosin (H&E), (*b*) Bielschowsky and (*c*) Masson's trichrome stains. Inset images: (*a*) red blood cells in the right hemiclitoris and muscle layer between the hemiclitores and cloaca; (*b*) nerves within the right hemiclitoris; (*c*) red blood cells and collagen within the right hemiclitoris. **C:** collagen; **CL**: cloaca; **HC**: hemiclitores; **M**: muscle cells; **N**: nerve fibres; **NB**: nucleated red blood cells; **SG**: scent glands. (Online version in colour.)

The hemiclitores were clearly identified in the tails of two female death adders, posterior to the cloaca and medial to the two scent glands (figure 2). DiceCT, dissection and histology revealed the hemiclitores as two independent structures, separated through the midline by connective tissue, that together form a triangular shape extending and tapering posteriorly (figures 2 and 3). The hemiclitores were prominent although small (figure 2; electronic supplementary material, table S1) and extended dorsally towards the spine. Like all other species examined, the hemiclitores lacked spines, sulcus spermaticus and retractor muscles, and could not be manually everted, unlike the adult and juvenile male death adders' hemipenes (electronic supplementary material, figure S1). Dissection and histology of female *A. antarcticus* revealed that each hemiclitoris had extensive erectile tissue that contained clusters of nucleated red blood cells in the numerous vascular spaces interwoven with collagen, which were identified by H&E and Trichrome stains (figure 3*a*,*c*). By contrast, the erectile tissue of the hemipenis had dense muscle fibres alongside but separate from collagen (electronic supplementary material, figure S1). Nerve bundles and single nerve strands were also present throughout the hemiclitores and hemipenes, as seen in the Bielschowsky silver stain (figure 3*b*; electronic supplementary material, figure S1*b*,*e*). The presence of erectile bodies with blood cells suggests that the hemiclitores engorge with blood, while the presence of abundant nerve bundles suggests that their stimulation may provide sensory feedback to the females.

### (c) Differentiating the hemiclitores and scent glands

To clear up the misidentification of scent glands with hemiclitores, i.e. [24], we investigated the DiceCT scan of *Lampropeltis abnorma* (figure 1*d*) and *A. antarcticus* (figure 2), and dissected a mature female *Morelia spilota* (figure 1*e*), which was one of the species used in [24]. We confirmed that the ‘ovoid structures cranial to the scent gland’ described by [24] were actually part of the scent gland because they clearly connect to the gland and extend to the cloacal opening (figures 1–3). Depending on where the tail was sliced longitudinally, it appeared as if the scent gland and duct were disconnected posteriorly, leading to misidentification of two individual ‘hemiclitores’ located posterior to the scent glands (figure 2). We confirmed that the structures labelled as ‘hemiclitores’ in [24] were actually ducts, by dissecting the tail in *M. spilota* and using a semi-blunt probe, we found the duct opening at the cloaca (figure 1*e*). This arrangement of hemiclitores medial to the scent gland and ducts was consistent across the females of the species examined (figures 1 and 2).

## 4. Discussion

Female genitalia are historically under-studied compared to males [3,4], and this neglect has delayed our understanding of reproductive biology and behaviour of females in nature. Even though the clitoris is present in most female amniotes [1], and as we demonstrate here, in snakes as well, very little is known about the possible functional role and evolution of the hemiclitores in squamates. Here, we report that the hemiclitores in snakes are diverse across a range of species and likely functional. These findings may help us broadly re-examine female choice in snakes via genital stimulation.

### (a) Evolutionary significance of snake hemiclitores

Our discovery of hemiclitores in snakes is timely in the field of reproductive biology given the recent enthusiasm for using innovative imaging techniques for explore female anatomy [1] and confusion surrounding the anatomy of hemipenes/hemiclitores in intersex snakes, which is stymieing progress in the field [36]. Quantifying morphological variation in hemiclitores among squamates will be important for understanding mating strategies and testing hypotheses of genital coevolution. The phenotypic diversity of hemiclitores is evident within and between families of snakes and lizards [36] and suggests that courtship and mating differences may have influenced the evolution of hemiclitores morphology. A future comparative study including more reproductively diverse species would help to elucidate the potential role(s) of the squamate hemiclitores.

Our discovery of well-developed, non-eversible hemiclitores in female adult snakes has previously not been accurately described and provides supporting evidence that hemiclitores have been retained across squamates. Several important differences between the male and female genitalia, and notable diversity of hemiclitores across species, challenge previous statements that squamate hemiclitores are a vestigial form of hemipenes, or an intersex hemipene [16], reviewed in [36]. The interspecific diversity of snake hemiclitores parallels that of the male hemipenes [37,38], suggesting that similar selection pressures may influence the shape, size and characteristics, such as detached pockets (figure 1*f*,*g*), of the hemiclitores. Further descriptions of hemiclitores, the vagina and conspecific male hemipenes morphology across snake species with different reproductive strategies will be important for mapping the full phenotypic variation and understanding genital evolution in squamate reptiles [1]. Moreover, variation in hemiclitores morphology presents new taxonomic characteristics that may prove useful for resolving the origin of snakes within other squamates (reviewed in [37,38]).

### (b) Functional significance of snake hemiclitores

To establish potential function of the hemiclitores, we look at diversity across species, where variation could indicate the action of selection. We investigated variation in gross hemiclitores morphology across clades spanning 100 Myr of snake evolution and found variation across pythonids, colubrids and viperids, and even variation among closely related elapids. The viperid and colubrid species presented with similar interspecific hemiclitores shape and size within each family (figure 1*a*–*d*), whereas elapids presented with significant interspecific variation in size, shape and characteristics such as detached pockets (figure 1*f*–*h* and figure 2). Characteristics, such as soft tissue detached pockets in *Pseudechis*, indicate that there are species groupings that may be comparable to taxonomic groupings based on hemipenis morphology and ornamentation, such as spines and hooks, and should be investigated. Additionally, these pockets might represent the ‘mere shallow invaginations' referenced in early descriptions of female squamate genitalia [39]. Unlike ‘pockets’ previously described from inverted intersex hemipenes in snakes [40], these pockets are not the result of inverted genital structures, but rather a pouch of soft tissue detached from the hemiclitores. The presence/absence of these pockets may aid in external access for the males to the anterior section of the hemiclitores in some species, but the function of this structure should be investigated further.

While hemipenes and hemiclitores in snakes share the same developmental pathways during embryogenesis [23,36], our histological comparison of these structures in *A. antarcticus* identified several anatomical differences between them (figure 3; electronic supplementary material, figure S1). The snake hemiclitores are composed of collagen and vascularized spaces (erectile tissue), connective tissue and dense innervation, but lack muscle fibres in the erectile tissue, and other hemipenis characteristics, such as spines. Since hemipenis spines and muscle fibres in the erectile tissue are present in both juvenile and adult males (electronic supplementary material, figure S1), it is unlikely that we missed their presence in our sample of females due to sexual immaturity or an early stage of genital development. Muscle fibres within the hemipenes provide structural support for inflation during hemipenile eversion, and the retractor muscles attached to the hemipenes allow retraction of the hemipenes back into the tail (electronic supplementary material, figure S1) [8]. A lack of these structures in the hemiclitores supports the observation that the hemiclitores are non-eversible in snakes, unlike hemiclitores in lizards [17–20]. Additionally, the hemiclitores are composed of erectile tissue that is likely to swell but not evert (e.g. **[**8]). Lizard hemipenes and hemiclitores both have muscle fibres and spines, and while these features are often present in snake hemipenes, they are absent in all the hemiclitores examined.

The presence of nerve bundles and single nerve fibres in the hemiclitores may be indicative of tactile sensitivity, similar to the mammalian clitoris [8]. The innervation and erectile tissue of the hemiclitores, and their position close to the posterior lip of the cloaca where the skin is thinner, could allow stimulation during mating through copulatory behaviours, such as tail wrapping and dorsal body looping [8,12,17–20]. These male mating behaviours could provide female sensory stimulation that may elicit female receptivity. The presence of erectile tissue with some evident blood cells suggests that the hemiclitores may have the ability to engorge with blood if stimulated, much like what has been observed in mammals (e.g. [10]), and other amniotes during sexual activity (e.g. [5]). However, the neurophysiology and density of these nerves in snake hemiclitores needs further investigation with more comprehensive histology/immunohistology and behavioural studies to determine whether they have a copulatory purpose [20].

### (c) Intersex hemiclitores or intersex hemipenes?

The literature on hemiclitores in snakes has suffered from either misinterpretation or misidentification with intersex genital anatomy [25–29,36,41]. Our anatomical description of hemiclitores in female snakes show that the ‘intersex hemiclitores' from previous studies are more accurately termed as ‘intersex hemipenes’. This is because early reports of intersexuality in snakes describe this condition as the presence of internal female characteristics (i.e. oviducts) alongside genitalia that are paired eversible uni- or bilobed structures with a sulcus spermaticus through the midline and retractor muscles [17,25,42]. Thus, intersex genitalia more closely resemble male hemipenes, albeit they are often a smaller size with minimal spine development. To our knowledge, intersex hemiclitores (accompanied by typical male gonads) have not previously been described. However, it is possible that intersex individuals with typical male gonads and hemiclitores exist, but their genitalia were not fully examined or are confused with small hemipenes. For example, Hoge [25] mentions that four *Bothrops insularis* embryos had testes with no hemipenes; however, the potential of intersex non-eversible hemiclitores was not investigated. Our description of hemiclitores morphology will allow future studies to properly assign genital characteristics of the hemiclitores and the hemipenes in squamates, which can result in better investigation of the prevalence of intersexual variation. Properly classifying intersex individuals according to whether they have testes and hemiclitores, or ovaries and hemipenes, would be the first step to potentially investigating the mechanisms that make intersex common in snakes.

## 5. Conclusion

Our study opens fruitful avenues for research into genital development, function and evolution. Our discovery of likely functional snake hemiclitores implies greater morphological diversity of genitalia within squamates than previously described, from the evertable lizard hemiclitores and squamate hemipenes to the non-eversible snake hemiclitores. Variation in the snake hemiclitores might prove to be correlated with courtship and mating behaviours and help us understand female choice. We suggest that the hemiclitores transduce sensation to the female snake during courtship and copulation, which might promote longer and more frequent mating leading to increased fertilization success. Further investigation into the sensory features of snake hemiclitores and hemipenes are needed to determine potential tactile sensitivity. Comparative morphological investigations of hemiclitores and hemipenes within and among taxa would also provide insight into the possible coevolution of male and female genitalia.

## Data Availability

The datasets supporting this article have been uploaded as part of the supplementary material and online from the Dryad Digital Respository: https://doi.org/10.5061/dryad.j6q573nh3 [43]. The data are provided in the electronic supplementary material [44].
